# Quantitative Determination of the Multicomponents With Overlapping Ultraviolet Spectra Using Wavelet-Packed Transform and Partial Least Squares

**DOI:** 10.1155/JAMMC/2006/86989

**Published:** 2006

**Authors:** Ling Gao, Shouxin Ren

**Affiliations:** 1 Department of Chemistry Inner Mongolia University Huhehot Inner Mongolia 010021 China

## Abstract

This paper presented a novel method named wavelet packet transform-based partial least squares method (WPTPLS) for simultaneous spectrophotometric determination of α-naphthylamine, p-nitroaniline, and benzidine. Wavelet packet representations of signals provided a local time-frequency description and separation ability between information and noise. The quality of the noise removal can be improved by using best-basis algorithm and thresholding operation. Partial least squares (PLS) method uses both the response and concentration information to enhance its ability of prediction. In this case, by optimization, wavelet function and decomposition level for WPTPLS method were selected as Db16 and 3, respectively. The relative standard errors of prediction (RSEP) for all components with WPTPLS and PLS were 2.23% and 2.71%, respectively. Experimental results showed WPTPLS method to be successful and better than PLS.


Hindawi Publishing Corporation
Journal of Automated Methods and Management in Chemistry
Volume 2006, Article ID 86989, Pages 1–6
DOI 10.1155/JAMMC/2006/86989

### Quantitative Determination of the Multicomponents with Overlapping Ultraviolet Spectra Using Wavelet-Packed Transform and Partial Least Squares

**Ling Gao and Shouxin Ren**

*Department of Chemistry, Inner Mongolia University, Huhehot 010021, Inner Mongolia, China*

Received 16 September 2004; Accepted 13 June 2005

This paper presented a novel method named wavelet packet transform-based partial least squares method (WPTPLS) for simultaneous spectrophotometric determination of $\alpha$-naphthylamine, p-nitroaniline, and benzidine. Wavelet packet representations of signals provided a local time-frequency description and separation ability between information and noise. The quality of the noise removal can be improved by using best-basis algorithm and thresholding operation. Partial least squares (PLS) method uses both the response and concentration information to enhance its ability of prediction. In this case, by optimization, wavelet function and decomposition level for WPTPLS method were selected as Db16 and 3, respectively. The relative standard errors of prediction (RSEP) for all components with WPTPLS and PLS were 2.23% and 2.71%, respectively. Experimental results showed WPTPLS method to be successful and better than PLS.

Copyright © 2006 L. Gao and S. Ren. This is an open access article distributed under the Creative Commons Attribution License, which permits unrestricted use, distribution, and reproduction in any medium, provided the original work is properly cited.

### 1. INTRODUCTION

Many efforts have been made in order to resolve overlapping signals in spectrophotometry. As a consequence of peak overlapping, the quality of analytical information is lower than what is derived from isolated peaks; the extent of the loss depends on the extent of overlap. In complex samples, however, spectral overlap is often occurring. Strongly overlapped signals do not permit direct determination by traditional methods without previous separation. To overcome this difficulty, multivariate analysis [1–3] such as partial least squares (PLS) and principal components regression (PCR), and so forth have been proved to be useful. When PLS and PCR methods were applied to analyze samples, the first problem encountered is to determine the number of components that they contain. Unfortunately, data obtained from instrumental measurements can be contaminated by noise. The presence of noise often causes overestimating the true number of chemical components. In order to eliminate noise, wavelet packet (WP) denoising method was used as a preprocessing step. Wavelet packet transform (WPT) is an important extension of wavelet transform (WT). WT is a powerful tool with a very rich mathematical content and great potential for application [4, 5]. WPT inherits the property of having a sparse representation of the original signal and time-frequency localization, and offers more flexibility than wavelet analysis [6, 7]. A novel approach tried here is to combine WPT with PLS to eliminate noise and improve the quality of regression. Aniline-type compounds are widely applied in industries such as chemistry, printing, and pharmacy, and are one of the most important raw materials for synthetic medicine, dye, insecticides, polymer, and explosives. Aniline-type compounds are highly poisonous, and can also cause cancer. Therefore, it is very important to test and analyze aniline-type compounds in environmental samples. Simultaneous determination of aniline-type compounds is very difficult due to their overlapping spectra. In this paper, WPTPLS method was developed and used to perform simultaneous determination of $\alpha$-naphthylamine, p-nitroaniline, and benzidine. Experimental results showed the proposed method to be successful and better than PLS.

### 2. THEORY

### 2.1. *WPT denoising*

A wavelet packet $W_{jnk}$ is generated from the base function

$$W_{jnk}(x) = 2^{-j/2} W_n(2^{-j}x - k), \tag{1}$$

where indices $j, n, k$ are the scale, the oscillation, and the localization parameter, respectively. $j, k \in Z$, $Z$ means the set of integers, $n = 0, 1, 2, \ldots, 2^j - 1$. The discrete wavelet transform (DWT) can be implemented by means of Mallat's pyramid algorithm [8]. DWT can be characterized as a recursive application of the high-pass and low-pass filters that form a quadrature mirror filter (QMF) pair. The theoretical background about DWT has been described in details [9]. The difference between WT and WPT is the decomposition path. In WPT, both the approximations and details are analyzed. The recursion is simply to filter and downsample all output of the previous level. A fast wavelet packet transform (FWPT) is expressed as

$$W_{j+1\,2n} = HW_{jn}, \qquad W_{j+1\,2n+1} = GW_{jn}, \tag{2}$$

where $W_{0,0}$ indicates the measured signal $f$, $H = \{h_l\}_{l\in Z}$ and $G = \{g_l\}_{l\in Z}$ are the low-pass and high-pass filters matrices. The first and second indices of $W$ indicate the level of decomposition and its position at that level. The reconstruction can be implemented by

$$W_{jn} = H^* W_{j+1\,2n} + G^* W_{j+1\,2n+1}, \tag{3}$$

where $H^*$ and $G^*$ represent the conjugate matrices of $H$ and $G$.

The wavelet packet denoising procedures include four steps: (1) WPT, (2) estimation of the best basis, (3) thresholding of wavelet packet coefficients, and (4) reconstruction. The best basis is selected according to entropy-based criterion proposed by Coifman and Wickerhauser [6]. Shannon entropy was applied in this case. The thresholding operation is implemented by the SURE method proposed by Donoho [10] based on Stein's unbiased risk estimation.

### 2.2. The wavelet packet transform partial least squares method

In the method, WPT is used as a tool for removing noise from original data. The denoising is applied to the wavelet packet domain as described above, prior to backtransforming it to original domain. The reconstructed matrices from standard and unknown mixtures were obtained for further PLS operation. The PLS algorithm is built on the properties of the nonlinear iterative partial least squares (NIPALS) algorithm by calculating one latent vector at a time. The NIPALS-PLS algorithm and calculating details were described previously [11].

According to this algorithm, the program called PWPT-PLS was designed to perform data compression and denoising as well as simultaneous determination.

### 3. EXPERIMENTAL

### 3.1. Apparatus and reagents

The Shimadzu UV-240 spectrophotometer furnished with OPI-2 function was used for all experiments; a legend Pentium IV microcomputer was used for all the calculations; pH measurements were made by a pH-3B digital pH-meter with a glass-saturated calomel dual electrode. All reagents were of analytical reagent grade. The water used was doubly distilled and deionized. Stock standard solutions of 2.000 mgml$^{-1}$ $\alpha$-naphthylamine, p-nitroaniline, and benzidine were prepared from correspondent reagents with water as solvents. Standard solutions were then prepared from their stock standard solutions by serial dilution as required. Acetic acid (HAc)-sodium acetate (NaAc) buffer solution (pH 6.30) was used.

### 3.2. Procedures

A series of mixed standard solutions containing various ratios of the three kinds of organic compounds was prepared in 25 ml standard flasks, 10.00 ml of HAc-NaAc buffer solution (pH 6.30) was added, and dilution with distilled water to mark. A blank solution was prepared similarly. Spectra were measured in 1 cm cuvettes between 250 nm and 460 nm at 2 nm intervals with respect to a reagent blank. An absorption matrix $D$ was built up. All the values measured were means of three replicate.

### 3.3. Evaluation of the performance of the test methods

Absolute and relative standard errors of prediction (SEP and RSEP) were used as the criteria for comparing the performances of the test methods. The SEP for a single component is given by (4); that for all components by (5). The RSEP is given by (6) [12]:

$$\text{SEP} = \sqrt{\frac{\sum_{j=1}^{m}\{C_{ij} - \hat{C}_{ij}\}^2}{m}}, \tag{4}$$

$$\text{SEP} = \sqrt{\frac{\sum_{i=1}^{n}\sum_{j=1}^{m}\{C_{ij} - \hat{C}_{ij}\}^2}{nm}}, \tag{5}$$

$$\text{RSEP} = \sqrt{\frac{\sum_{i=1}^{n}\sum_{j=1}^{m}\{C_{ij} - \hat{C}_{ij}\}^2}{\sum_{i=1}^{n}\sum_{j=1}^{m}C_{ij}^2}}, \tag{6}$$

where $C_{ij}$ and $\hat{C}_{ij}$ are the actual and estimated concentrations, respectively, for the $i$th component in the $j$th mixture, $m$ is the number of mixtures, and $n$ is the number of components.

### 4. RESULTS AND DISCUSSIONS

### 4.1. Absorbance spectra of the system

Figure 1 shows the absorption spectra of p-nitroaniline $\alpha$-naphthylamine, and benzidine and their mixed solution. The maximum absorptions of three components were 380 nm,

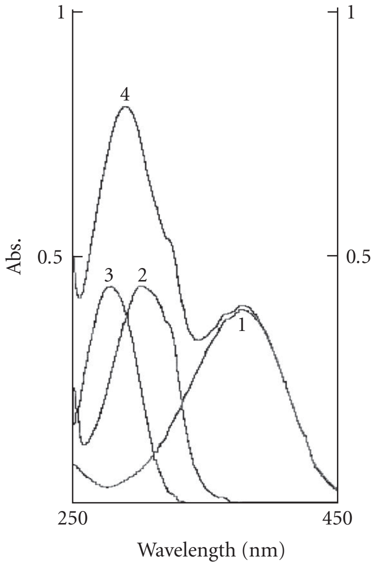

Figure 1: The absorpation spectra 4.000 $\mu$gml$^{-1}$ p-nitroaniline (1); 12.00 $\mu$gml$^{-1}\alpha$-naphthylamine (2); 3.200 $\mu$gml$^{-1}$ benzidine (3), and their mixture compounds (4).

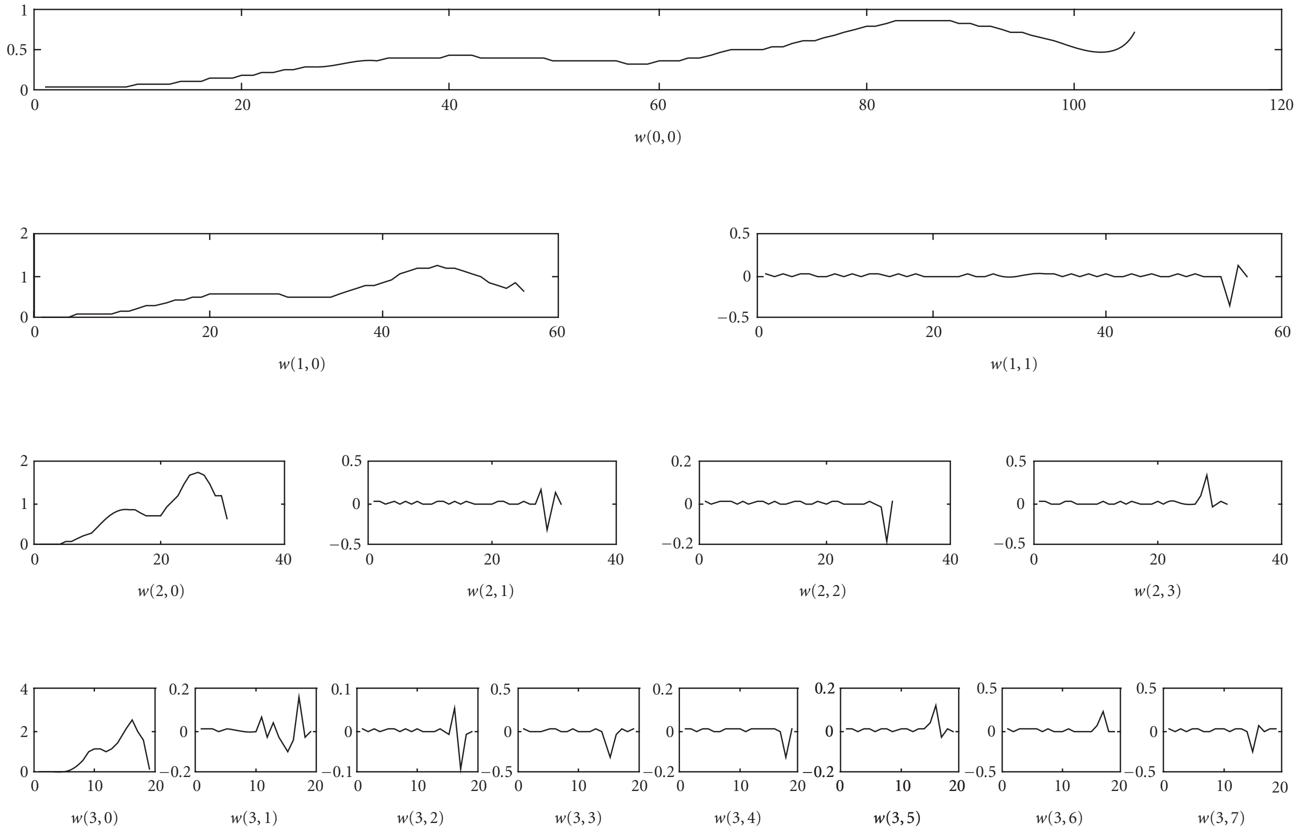

Figure 2: The parts of WP coefficients obtained by wavelet packet transform.

302 nm, and 278 nm, respectively. It can be seen from Figure 1 that the absorption spectra of three components exhibited are seriously overlapped in their absorbing regions, so that for mixed solution only one peak can be recognized.

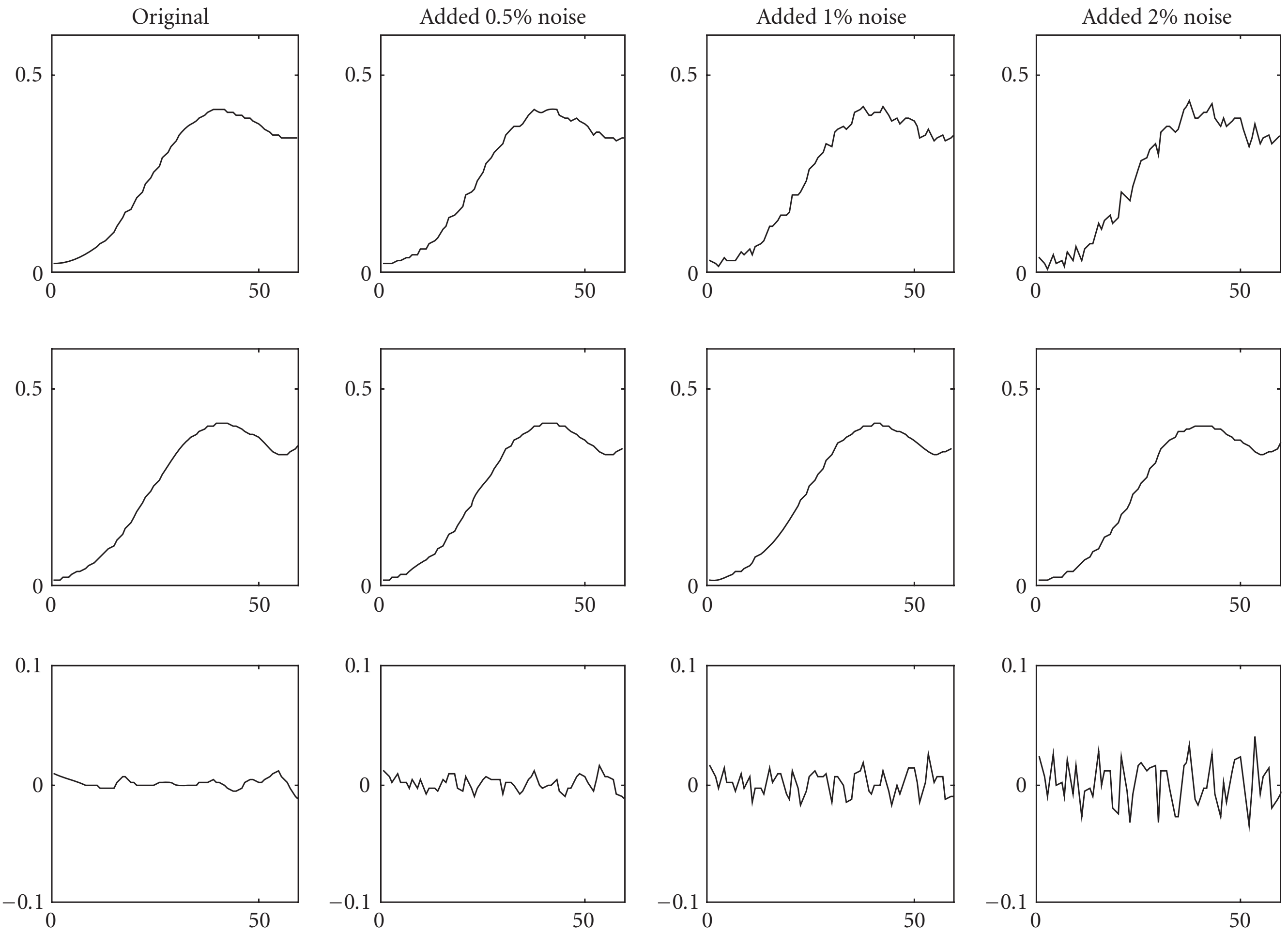

Figure 3: Original raw spectra (row 1), reconstructed spectra (row 2), and their difference obtained by means of WP denoising (row 3) for different amounts of noise added (indicated at the head of columns).

### 4.2. Wavelet packet transform and wavelet packet denoising

Here, we selected mean spectra of $D$ matrix as original signal $f$. WPT of the signal $f$ was carried out using FWPT algorithm. The part of WP coefficients obtained by FWPT is shown in Figure 2. Each coefficient is identified by the couple of index $(j, n)$, where $j$ is the level of decomposition and $n$ is the position at that level. From Figure 2, it is obvious that the $w(j, 0)$ only contains a positive part and is similar to the original signal. The others are composed of both positive and negative parts. Each block of the coefficients describes the components of the signal $f$ related to a certain frequency band. This flexible time-frequency resolution enables the WP to characterize locally the most relevant parts of a signal and hence to adequately represent a signal with relatively small number of coefficients. In the spectrophotometric measurements, the analytical signals usually center in low-frequency part, whereas the noise in high-frequency part. The aim of WP denoising is to extract the desired signal from a complex instrument output, where the signal is present along with noise. Random Gaussian noise was added to the mean spectra for assessing the WP denoising method. Original and reconstructed spectra as well as their difference with different added noise are displayed in Figure 3. It was found that WPT provides an appropriate approach for denoising even in case where 2% noise is added. Thus the method is safe for preprocessing two-dimensional raw data matrix in the following WPTPLS operation.

### 4.3. The wavelet packet transform partial least squares method

Each of the wavelet functions has different characteristics. The wavelet function, which is optimal for a given signal, is not necessarily the best for another type of signal. Thus, the choice of the wavelet functions is very important for this technique. In this work, the wavelet functions tested were Coiflet $1, 2, \ldots, 5$, Daubechies $4, 6, \ldots, 20$, Symmlet $4, 5, \ldots, 8$. It is possible to use the predictive parameters SEP and RSEP to find the optimum choice of functions. In similar way, one-to-six decomposition levels $L$ were tested

Table 1: Optimization of wavelet functions.

| Wavelet function | SEP ( $\mu$gml$^{-1}$) | RSEP (%) |
|---|---|---|
| Daubechies 2 | 2.62 | 0.227 |
| Daubechies 4 | 2.66 | 0.230 |
| Daubechies 6 | 2.70 | 0.233 |
| Daubechies 8 | 2.76 | 0.239 |
| Daubechies 10 | 2.52 | 0.217 |
| Daubechies 12 | 2.57 | 0.222 |
| Daubechies 14 | 2.41 | 0.208 |
| Daubechies 16 | 2.23 | 0.192 |
| Daubechies 18 | 2.47 | 0.214 |
| Daubechies 20 | 2.43 | 0.210 |
| Symmlet 5 | 2.71 | 0.234 |
| Symmlet 6 | 2.72 | 0.235 |
| Symmlet 7 | 2.75 | 0.238 |
| Symmlet 8 | 2.71 | 0.234 |
| Coiflet 1 | 3.35 | 0.289 |
| Coiflet 2 | 2.73 | 0.235 |
| Coiflet 3 | 2.72 | 0.235 |
| Coiflet 4 | 2.69 | 0.232 |
| Coiflet 5 | 2.74 | 0.237 |

too. The influence of wavelet functions and decomposition levels is listed in Tables 1 and 2. According to these experimental results, wavelet functions and decomposition level for WPTPLS method were selected as Daubechies (Db) 16 and 3.

A training set of 16 samples formed by the mixture of the three organic compounds was designed according to four-level orthogonal array design with the $L_{16}(4^5)$ matrix. Table 3 summarizes the composition of the training set. The experimental data obtained from the training set were arranged in matrix $D$, where each column corresponds the absorbance of different mixtures at a given wavelength and each row represents the spectrum obtained at a given mixture. With FWPT, one can treat each spectrum at a given mixture. Therefore, in the same way each row vector of matrix $D$ and $D_u$ was decomposed, and denoised by best-basis selection and thresholding operation, then reconstructed by applying inverse FWPT. Determining the number of factors is one of the most important steps in PLS method. The essence of the step is the pseudorank determination of the raw experimental data. Three principal factors for the case were selected based on previously reported methods [13]. Before starting the WPTPLS calculation, mean centering and data standardization were performed as preprocessing. After this transform, the matrix where each column had zero mean and a variance equals to the unity was obtained. Using program PWPTPLS, the concentrations of the three organic compounds for a test set were calculated. Actual concentrations, found concentrations, and their recoveries are listed in Table 4. The experimental results showed that the SEP and RSEP for all components were 0.192 $\mu$gml$^{-1}$ and 2.23%.

Table 2: Optimization of wavelet decomposition level.

| L | SEP ( $\mu$gml$^{-1}$) | RSEP (%) |
|---|---|---|
| 1 | 0.234 | 2.71 |
| 2 | 0.236 | 2.74 |
| 3 | 0.230 | 2.66 |
| 4 | 0.302 | 3.49 |
| 5 | 1.39 | 16.1 |
| 6 | 1.95 | 22.6 |

Table 3: Composition of the training set.

| Sample no. | Concentration ($\mu$gml$^{-1}$) | | |
|---|---|---|---|
| | I | II | III |
| 1 | 0.8000 | 2.000 | 0.4000 |
| 2 | 0.8000 | 4.000 | 2.4000 |
| 3 | 0.8000 | 16.00 | 5.600 |
| 4 | 0.8000 | 24.00 | 8.000 |
| 5 | 2.400 | 2.000 | 2.400 |
| 6 | 2.400 | 4.000 | 0.4000 |
| 7 | 2.400 | 16.00 | 8.000 |
| 8 | 2.400 | 24.00 | 5.600 |
| 9 | 5.600 | 2.000 | 5.600 |
| 10 | 5.600 | 4.000 | 8.000 |
| 11 | 5.600 | 16.00 | 0.4000 |
| 12 | 5.600 | 24.00 | 2.400 |
| 13 | 8.000 | 2.000 | 2.400 |
| 14 | 8.000 | 4.000 | 8.000 |
| 15 | 8.000 | 16.00 | 5.600 |
| 16 | 8.000 | 24.00 | 0.4000 |

I: p-nitroaniline, II: $\alpha$-naphthylamine, III: benzidine.

### *4.4. A comparison of WPTPLS and PLS*

In order to evaluate WPTPLS method, two methods were tested in the study with a set of synthetic unknown samples. The RSEP for the two methods are given in Table 5. The RSEP for all components calculated by WPTPLS and PLS methods were 2.23% and 2.71%, respectively. The results demonstrated that the WPTPLS method had better performance than PLS method.

### 5. CONCLUSIONS

A method named WPTPLS was developed for multicomponent spectrophotometric determination. The method combines the idea of the WPT denoising with PLS regression for enhancing noise removal ability and quality of the regression. In WP denoising the time-frequency localization, best-basis algorithm and thresholding operation were used to improve the quality of denoising. In PLS operation, errors both in the concentration and spectra were taken into account to improve predictive properties. Experimental results show the clear superiority of WPTPLS over PLS method.

Table 4: Actual concentration, found concentration, and percentage recovery of the synthetic unknowns.

| Sample no. | Actual concentration ($\mu$gml$^{-1}$) | | | Found concentration ($\mu$gml$^{-1}$) WPTPLS | | | Found concentration ($\mu$gml$^{-1}$) PLS | | | Recovery (%) WPTPLS | | | Recovery (%) PLS | | |
|---|---|---|---|---|---|---|---|---|---|---|---|---|---|---|---|
| | I | II | III | I | II | III | I | II | III | I | II | III | I | II | III |
| 1 | 1.600 | 3.200 | 0.8000 | 1.758 | 3.474 | 0.822 | 1.760 | 3.379 | 0.8377 | 109.9 | 108.6 | 102.7 | 110.0 | 105.6 | 104.7 |
| 2 | 1.600 | 12.00 | 4.000 | 1.547 | 12.27 | 3.975 | 1.541 | 12.48 | 3.951 | 96.7 | 102.2 | 99.4 | 96.3 | 104.0 | 98.8 |
| 3 | 1.600 | 20.00 | 6.400 | 1.543 | 19.91 | 6.433 | 1.556 | 19.66 | 6.460 | 96.4 | 99.5 | 100.5 | 97.2 | 98.3 | 100.9 |
| 4 | 4.000 | 3.200 | 4.000 | 3.863 | 2.790 | 4.121 | 3.866 | 2.722 | 4.131 | 96.6 | 87.2 | 103.0 | 96.6 | 85.1 | 103.3 |
| 5 | 4.000 | 12.00 | 6.400 | 3.987 | 12.57 | 6.317 | 3.980 | 12.75 | 6.295 | 99.7 | 104.8 | 98.7 | 99.5 | 106.3 | 98.4 |
| 6 | 4.000 | 20.00 | 0.8000 | 4.036 | 19.75 | 0.7501 | 4.033 | 19.84 | 0.7404 | 100.9 | 98.7 | 93.8 | 100.8 | 99.2 | 92.5 |
| 7 | 6.400 | 3.200 | 6.400 | 6.458 | 2.961 | 6.495 | 6.460 | 2.866 | 6.501 | 100.9 | 92.5 | 101.5 | 100.9 | 89.6 | 101.6 |
| 8 | 6.400 | 12.00 | 0.8000 | 6.323 | 12.16 | 0.6775 | 6.324 | 12.09 | 0.6885 | 98.8 | 101.3 | 84.7 | 98.8 | 100.7 | 86.1 |
| 9 | 6.400 | 20.00 | 4.000 | 6.484 | 19.71 | 4.009 | 6.480 | 19.82 | 3.995 | 101.3 | 98.6 | 100.2 | 101.3 | 99.1 | 99.9 |

I: p-nitroaniline, II: $\alpha$-naphthylamine, III: benzidine.

Table 5: SEP and RSEP values for organic compounds system by two methods.

| Methods | SEP ($\mu$gml$^{-1}$) | | | | RSEP (%) | | | |
|---|---|---|---|---|---|---|---|---|
| | I | II | III | Total components | I | II | III | Total components |
| WPTPLS | 0.0866 | 0.313 | 0.0749 | 0.192 | 1.94 | 2.30 | 1.71 | 2.23 |
| PLS | 0.0859 | 0.387 | 0.0829 | 0.234 | 1.93 | 2.84 | 1.89 | 2.71 |

I: p-nitroaniline, II: $\alpha$-naphthylamine, III: benzidine.

### ACKNOWLEDGMENT

The authors would like to thank National Natural Science Foundation of China and Natural Science Foundation of Inner Mongolia for financial support of this project.

### REFERENCES

[1] S. Wold, M. Sjostrom, and L. Eriksson, "PLS-regression: a basic tool of chemometrics," *Chemometrics and Intelligent Laboratory Systems*, vol. 58, no. 2, pp. 109–130, 2001.

[2] F. Yacoub and J. F. MacGregor, "Product optimization and control in the latent variable space of nonlinear PLS models," *Chemometrics and Intelligent Laboratory Systems*, vol. 70, no. 1, pp. 63–74, 2004.

[3] E. P. Tsaousoglou, S. D. Bolis, and C. E. Efstathiou, "Monte Carlo simulation for the prediction of precision of absorbance measurements with a miniature CCD spectrometer," *Journal of Automated Methods & Management in Chemistry*, vol. 25, no. 2, pp. 35–42, 2003.

[4] I. Daubechies, "Orthogonal bases of compactly supported wavelets," *Communications on Pure and Applied Mathematics*, vol. 41, pp. 909–996, 1988.

[5] G. Beylkin, "On the representation of operators in bases of compactly supported wavelets," *SIAM Journal on Numerical Analysis*, vol. 29, no. 6, pp. 1716–1740, 1992.

[6] R. R. Coifman and M. V. Wickerhauser, "Entropy-based algorithms for best basis selection," *IEEE Transactions on Information Theory*, vol. 38, no. 2, pp. 713–718, 1992.

[7] B. Jawerth and W. Sweldens, "An overview of wavelet based multiresolution analyses," *SIAM Review*, vol. 36, no. 3, pp. 377–412, 1994.

[8] S. G. Mallat, "Multiresolution approximations and wavelet orthonormal bases of $L^2(\mathbf{R})$," *Transactions of the American Mathematical Society*, vol. 315, no. 1, pp. 69–87, 1989.

[9] S. Ren and L. Gao, "Simultaneous quantitative analysis of overlapping spectrophotometric signals using wavelet multiresolution analysis and partial least squares," *Talanta*, vol. 50, no. 6, pp. 1163–1173, 2000.

[10] D. L. Donoho, "De-noising by soft-thresholding," *IEEE Transactions on Information Theory*, vol. 41, no. 3, pp. 613–627, 1995.

[11] L. Gao and S. Ren, "Simultaneous spectrometric determination of manganese, zinc and cobalt by Kernel partial least-squares method," *Journal of Automatic Chemistry*, vol. 20, no. 6, pp. 179–183, 1998.

[12] M. Otto and W. Wegscheider, "Spectrophotometric multicomponent analysis applied to trace metal determinations," *Analytical Chemistry*, vol. 57, no. 1, pp. 63–69, 1985.

[13] S. Ren and L. Gao, "Simultaneous spectrophotometric determination of copper (II), lead (II), and cadmium (II)," *Journal of Automatic Chemistry*, vol. 17, no. 3, pp. 115–118, 1995.

